# The clinical value of daily routine chest radiographs in a mixed medical–surgical intensive care unit is low

**DOI:** 10.1186/cc3955

**Published:** 2005-12-30

**Authors:** Marleen E Graat, Goda Choi, Esther K Wolthuis, Johanna C Korevaar, Peter E Spronk, Jaap Stoker, Margreeth B Vroom, Marcus J Schultz

**Affiliations:** 1 Medical student, Department of Intensive Care Medicine, Academic Medical Center, University of Amsterdam, Amsterdam, The Netherlands; 2 Resident, Departments of Intensive Care Medicine and Internal Medicine, Academic Medical Center, University of Amsterdam, Amsterdam, The Netherlands; 3 Resident, Departments of Intensive Care Medicine and Anesthesiology, Academic Medical Center, University of Amsterdam, Amsterdam, The Netherlands; 4 Clinical Epidemiologist, Department of Clinical Epidemiology and Biostatistics, Academic Medical Center, University of Amsterdam, Amsterdam, The Netherlands; 5 Internist-intensivist, Department of Intensive Care Medicine, Gelre Hospital (Location Lukas), Apeldoorn, The Netherlands; 6 Radiologist, Department of Radiology, Academic Medical Center, University of Amsterdam, Amsterdam, The Netherlands; 7 Anaesthsiologist-intensivist, Department of Intensive Care Medicine, Academic Medical Center, University of Amsterdam, Amsterdam, The Netherlands; 8 Internist-intensivist, Research Coordinator, Department of Intensive Care Medicine, Academic Medical Center, University of Amsterdam, Amsterdam, The Netherlands

## Abstract

**Introduction:**

The clinical value of daily routine chest radiographs (CXRs) in critically ill patients is unknown. We conducted this study to evaluate how frequently unexpected predefined major abnormalities are identified with daily routine CXRs, and how often these findings lead to a change in care for intensive care unit (ICU) patients.

**Method:**

This was a prospective observational study conducted in a 28-bed, mixed medical–surgical ICU of a university hospital.

**Results:**

Over a 5-month period, 2,457 daily routine CXRs were done in 754 consecutive ICU patients. The majority of these CXRs did not reveal any new predefined major finding. In only 5.8% of daily routine CXRs (14.3% of patients) was one or more new and unexpected abnormality encountered, including large atelectases (24 times in 20 patients), large infiltrates (23 in 22), severe pulmonary congestion (29 in 25), severe pleural effusion (13 in 13), pneumothorax/pneumomediastinum (14 in 13), and malposition of the orotracheal tube (32 in 26). Fewer than half of the CXRs with a new and unexpected finding were ultimately clinically relevant; in only 2.2% of all daily routine CXRs (6.4% of patients) did these radiologic abnormalities result in a change to therapy. Subgroup analysis revealed no differences between medical and surgical patients with regard to the incidence of new and unexpected findings on daily routine CXRs and the effect of new and unexpected CXR findings on daily care.

**Conclusion:**

In the ICU, daily routine CXRs seldom reveal unexpected, clinically relevant abnormalities, and they rarely prompt action. We propose that this diagnostic examination be abandoned in ICU patients.

## Introduction

Chest radiographs (CXRs) are frequently obtained in intensive care units (ICUs) [1]. They can be obtained routinely, on a daily basis (so-called 'daily routine CXRs'); such radiographs are generally ordered without any specific reason. Another strategy is to order CXRs only if clinically indicated (so-called 'on demand CXRs'); these radiographs are usually obtained following a change in clinical status or supportive devices.

The consensus opinion of the American College of Radiology Expert Panel is that daily routine CXRs are indicated in patients with acute cardiopulmonary problems and in patients receiving mechanical ventilation [2]. In practice, this includes the majority of ICU patients. However, two different schools of thought exist on the utility of daily routine CXRs in ICUs. Although many ICU physicians adhere to consensus opinion mentioned above, stating that the incidence of abnormalities on daily routine CXRs is sufficiently high to justify ordering these radiographs [3-5], others suggest that these CXRs can safely be abandoned [6-11]. Interestingly, most studies on the efficacy of daily routine CXR did not attempt to discriminate between clinically relevant and irrelevant findings, and simply reported on all abnormalities [12]. At present, in many ICUs CXRs are still routinely obtained on a daily basis, at least in The Netherlands [13].

There may be advantages to eliminating daily routine CXRs. First, a routine strategy carries the risk that abnormalities that either are of little importance or represent false-positive findings may be acted upon. Second, substantial savings can be achieved by limiting the number of CXRs ordered in ICUs. Most importantly, it is not clear whether obtaining daily routine CXRs truly alters the daily management of ICU patients. Therefore, we conducted the present study to determine the incidence of major abnormalities on daily routine CXRs and their impact on management of ICU patients.

## Materials and methods

Data on all daily routine CXRs ordered at the ICU of the Academic Medical Center – a university hospital in The Netherlands – were prospectively collected and evaluated over a five month period. All data were entered into a computerized database (Microsoft Access 2003; Microsoft Inc., Richmond, VA, USA). CXRs from readmitted patients were excluded from the analysis. During the study period no attempt was made to alter the daily routine strategy. The study protocol was approved by the local ethics committee.

During the study period, daily routine CXRs were conducted between 08:00 hours and 09:00 hours each day. For each CXR performed, the subspecialty fellow, resident, or intern completed a specially developed data sheet, which was printed on the back of the normal CXR request form. On this data sheet clinically expected abnormalities, in addition to the indication for each CXR (for example, 'daily routine' or 'on demand') was documented. The attending physician ticked several options to indicate whether a certain finding was expected, and whether it was 'old' (for instance, already present on preceding CXRs) or 'new' (for instance, not present on preceding CXRs; the included expected abnormalities are summarized in Table 1). Collection of data started after a one month trial period, during which the scoring system was tested to see whether it was practical, and to ensure that all involved ICU physicians and radiologists completed the forms during the study period.

It was unit policy to obtain CXRs after insertion of endotracheal tubes, intravenous lines and chest drains, but not after insertion of nasogastric tubes. In addition, CXRs were obtained in the case of worsening of oxygenation. As a rule, no routine CXR was ordered if an on-demand CXR was ordered within the four hours before the morning round. In case a daily routine CXR was ordered but the attending physician, together with his or her supervisor, had developed a specific question about the performed CXR (for instance, if it were not obtained then an on-demand CXR would have been ordered), it was analyzed as though it were an on-demand CXR. Importantly, this change in categorization was only possible before any of the ICU physicians could see the CXR, in order to prevent bias.

All CXRs were interpreted by an independent radiologist on the day the CXR was performed. Similar to the ICU physicians, the radiologist structurally interpreted the CXR for each patient (for example, the radiologist ticked whether radiological abnormalities [summarized in Table 1] were absent or present and, if an abnormality was present, whether it was judged to be an 'old' or 'new' finding). In case an abnormality was worsening, and fulfilling the criteria as in table 1, it was categorized as 'new'. All CXRs were reviewed by the team at 10:00 hours, when the radiologist communicated any positive findings. The following definitions were used: a 'new expected finding' was any new finding that had been predicted by the attending physician; and 'old expected finding' was any old finding predicted by the attending physician; a 'new unexpected finding' was any new finding not predicted by the attending physician; and an 'old unexpected finding' was any old finding not expected by the attending physician.

If an important finding (as mentioned in Table 1) was found, then we determined whether any action was taken because of the new and unexpected finding. To do this, four of us (MG, GC, EW and MS) carefully read the medical records, checked the patient data management system (Metavision, iMDsoft, Sassenheim, The Netherlands) and searched the hospital information system for the following: orders for sputum cultures or performance of a bronchoalveolar lavage for culture, or start of or a change in antimicrobial therapy in case of unexpected infiltrates on the CXR; repositioning of tubes in case of malposition of orotracheal tubes (ignoring planned extubations); ultrasound of the thorax in case of pleural effusion on the CXR, start or change in medication (diuretics); insertion of a pleural drain; and repositioning of devices in the case of malposition of medical devices other than orotracheal tubes (ignoring planned changes such as removal of intravenous lines). The observers were not involved in the daily care of the patients, and ICU physicians were not aware of this part of the observation. As a consequence, the clinical relevance of the predefined abnormalities could not be evaluated in some cases, specifically in case of large atelectasis and severe pulmonary congestion.

Data were analyzed together for all patients combined as well as for separate patient groups (general surgery patients, neurosurgery patients, cardiothoracic surgery patients, medical patients, and other patients). The incidence of clinically important abnormalities was compared by χ^2 ^test using SPSS 11.5.1 software (SPSS Inc., Chicago, IL, USA). *P *< 0.05 was considered statistically significant.

## Results

During the five month period of study, 4,404 CXRs were obtained during 822 ICU admittances of 754 patients. Once CXRs of patients who were admitted more than once were excluded, 3,894 CXRs remained to be analyzed. Of these, 2,457 were categorized as daily routine CXRs (63.1%). No CXRs were requested without a completed data sheet. Demographic data and major admitting diagnoses for patients are presented in Table 2.

The majority of daily routine CXRs (94.2%) did not reveal any new and unexpected predefined abnormalities. Ninety-six of the daily routine CXRs showed an old and expected predefined abnormality (3.9%). Of the 19 new abnormalities expected by the ICU physicians, only 3 (15.8%) were actually found by the radiologists (Table 3). New and unexpected predefined abnormalities were found in a minority of daily routine CXRs (5.8%; Table 3). The most common unexpected abnormalities were malposition of the orotracheal tube (32 times in 26 patients), severe pulmonary congestion (29 in 25), large atelectases (24 in 20), large infiltrates (23 in 22), pneumothorax/pneumomediastinum (14 in 13), and severe pleural effusion (13 in 13; table 3). Fewer than half of the radiographs with a potentially clinically relevant abnormality resulted in action: in 14.3% of patients did daily routine CXRs exhibit an unexpected abnormality, and in 6.4% of patients did these radiologic abnormalities result in a change to therapy (Table 3).

Similarly, most of the daily routine CXRs that were re-categorized as on-demand CXRs (because the attending physician had developed a specific question about the already routinely obtained CXR) did not reveal any new and unexpected predefined abnormality (Table 4). Only 11 unexpected abnormalities were encountered that caused a change to therapy (11 patients; for example, large infiltrates [*n *= 1], severe pleural effusion [*n *= 1], pneumothorax [*n *= 3], and malposition of oropharyngeal tube [*n *= 1], central venous line [*n *= 3], or drain [*n *= 1]).

The sensitivity and specificity of the clinicians in predicting changes on daily routine CXR were 2.1% (3/145) and 99.3% (2296/2312), respectively. Although sensitivity improved with those CXRs that were categorized as on-demand CXRs (21.0% [8/38]), specificity dropped to 59% (167/283).

Subgroup analysis revealed no important differences between groups (Table 5). Only in neurosurgical patients was the yield of daily routine CXRs lower as compared with the other admittance category groups. Similarly, the number of daily routine CXRs with a new and unexpected abnormality resulting in a change to therapy was similar among groups.

## Discussion

The present study was performed to investigate the clinical value of daily routine CXRs in critically ill patients. We showed not only that the incidence of potentially clinically relevant abnormalities was low but also that more than half of these abnormalities did not influence daily management.

Although other studies found a high incidence of radiographic abnormalities on daily CXR (for review [12]), our study confirms the markedly lower incidence of radiographic abnormalities in studies that restricted the analysis to 'new and unexpected' abnormalities [6,14]. These studies were all relatively small, however. The present study is the largest study on this topic, not only with respect to the evaluated number of CXRs but also with respect to the number of patients.

Chahine-Malus and coworkers [9] reported previously in this journal on the utility of daily routine CXRs in clinical decision making in the ICU. In that study, a questionnaire was completed for each radiograph, addressing the indication for the radiograph and whether it changed the patient's management. Of the CXRs performed in the medical and surgical patients, 20% and 26%, respectively, would have led to one or more management changes. The majority of changes were related to an adjustment of an invasive device. Our findings are in accordance with those of this previous study, at least in part. Indeed, in our study most CXR-induced changes were simple adjustments to medical devices. Incidences of CXR-induced changes were noticeably lower in our study, however, which may be explained by the fact that physicians were not asked whether they would make changes in daily management of their patients in the present study; instead, we observed whether abnormalities on the CXRs led to a change in therapy. We believe that this is a more accurate way to determine the value of the daily routine CXR.

Several important drawbacks of the present study must be mentioned. The study design allowed daily routine CXRs to be recategorized as on-demand radiographs if the attending physician had developed a specific question about the already routinely obtained radiograph. Although this change in classification was only possible before the physicians had seen the CXR (for instance, before the results were revealed at the daily meeting with the radiologist), this practice might have caused bias. However, classifying these CXRs as daily routine radiographs instead of on-demand radiographs did not change the results. Radiologists were not blinded to the expectations of the clinical fellows, residents, or interns; radiologists were able to read the back of each request form. We did not wish to interfere with daily practice in the study, however. Finally, the present analysis did not evaluate whether the absence of abnormalities influenced daily management in our ICU. For instance, the absence of infiltrates in a patient with fever may prompt physicians to look for other infections, and the absence of radiological signs of pulmonary congestion might have resulted in another fluid therapy regimen.

We did not score for the clinical relevance of the unexpected presence of large atelectasis or severe pulmonary congestion. We opted not to evaluate these two abnormalities because we were uncertain whether we could adequately score for this in an unbiased manner. ICU patients receive diuretics every day for many reasons, not just because of the presence of pulmonary congestion. Similarly, physiotherapy and use of (higher) levels of positive end-expiratory pressure are applied routinely in our ICU, and are not related to the presence of abnormalities on the CXR. Unfortunately, these abnormalities formed a substantial part of all new and unexpected abnormalities in our analysis (1.0% and 1.2% of all daily routine CXRs showed these two findings). However, even if we assume that all daily routine CXRs that showed one of these findings would have resulted in a change to therapy, the value of this diagnostic tool remained low (for example, 4.8% of all daily routine CXRs would have resulted in a change to therapy).

Sensitivity of the physicians in predicting changes on daily routine CXRs was extremely low in our study. This was very much in contrast with findings reported by Bhagwanjee and Muckart [8], who found a sensitivity of 95% for two examiners for comparable abnormalities in a similar group of patients. This difference may very well result from differences in study design; in the study conducted by Bhagwanjee and Muckart two examiners carefully evaluated patients to look for abnormalities, whereas in the present study 'sensitivity' was probably based sometimes on little more than a proposition that an abnormality could be present, and did not represent a prediction based on thorough examination.

To date, only two studies have compared a daily routine strategy (in which CXRs were taken routinely every day as well as on clinical indication) with a restrictive strategy (in which CXRs were taken only if clinically indicated) [10,11]. Price and coworkers [10] showed that length of stay in ICU or hospital and duration of mechanical ventilation were not negatively influenced by the elimination of daily routine CXRs. This prospective, nonrandomized, controlled study was performed in a paediatric intensive care unit, however. In a prospective, randomized, observational study, Krivopal and coworkers [11] determined whether there was any difference in diagnostic, therapeutic and outcome efficacy between a routine and a nonroutine CXR strategy in mechanically ventilated medical patients. Like in the study conducted by Price and coworkers, there was no difference in length of stay in ICU or hospital and duration of mechanical ventilation between the two groups. Unfortunately, this study was small and probably underpowered.

## Conclusion

The impact of daily routine CXRs on clinical management in our mixed medical–surgical ICU was low. Based on the present analysis, we have decided to exclude daily routine CXRs from patient management.

## Key messages

• 	The diagnostic yield of daily routine CXR in a mixed medical–surgical ICU is low.

• 	The small impact of daily routine CXRs on clinical management of critically ill patients in a mixed medical–surgical ICU justifies elimination of this diagnostic test, but additional studies, specifically in centres with different case-mix, are necessary before these results can be generalized to all types of ICU.

## Abbreviations

CXR = chest radiograph; ICU = intensive care unit.

## Competing interests

The authors declare that they have no competing interests.

## Authors' contributions

MG, GC and EW participated in the collection and interpretation of the data and were involved in drafting the manuscript. MG participated in analysis and interpretation of the data and in drafting the manuscript. PS, JS and MV contributed to the conception and design of the study and manuscript revision. JK was involved in the design and statistical analysis of the study. MS conceived and coordinated the study and was involved in the interpretation of the data and manuscript revision. All authors read and approved the final manuscript.

## Figures and Tables

**Table 1:** Findings (expected) on daily routine chest radiographs for which ICU physicians and radiologist could score

Abnormality	Comments
Large atelectasis	≥2 lobes
Large infiltrates	≥1 lobe
'Severe' pulmonary congestion	
'Severe' pleural effusion	
Pneumothorax or pneumomediastinum	Any abnormal air collection
Malposition of oropharyngeal tube	<2 cm from carina or above stem cords
Malposition of intravenous lines	Tip in right atrium or outside lumen (pulmonary artery catheter: tip in right atrium), or change in position
Malposition of intra-aortic balloon pump	
Malposition of gastric tube	Tip outside the stomach
Malposition of drains	Displacement >5 cm or outside pleural space

Abnormalities were scored by residents or clinical fellows if expected, and – separately – by radiologist if present. In addition, both requesting physician and radiologist determined whether the (expected) finding was 'old' or 'new' (see text for details). ICU, intensive care unit.

**Table 2:** Demographic data

Parameter	Value
Total number of patients (*n*)	754
Age (years)	59.8 ± 15.9
Male (*n *[%])	475 (63.0)
Length of stay (days; median [IQR])	2.5 (1.5–5.5)
Mortality (%)	9.5
APACHE II score	16.5 ± 7.0
SAPS II score	38.4 ± 15.1
Reason for admission to the ICU (*n*)	
Medical	197
General surgery	144
Cardiopulmonary surgery	317
Neurosurgery	69
Other	27

Data are expressed as means ± standard deviation, unless stated otherwise. APACHE, Acute Physiology and Chronic Health Evaluation; ICU, intensive care unit; IQR, interquartile range; SAPS, Simplified Acute Physiology Score.

**Table 3:** Incidence of new expected and new unexpected predefined major abnormalities in 2,457 daily routine chest radiographs

Abnormalities	Expected abnormalities^a^		Unexpected abnormalities^a^	
	
	Abnormalities expected by the ICU physician	Abnormalities found by the radiologist	Unexpected abnormalities found by the radiologist	Abnormalities resulting in a change in therapy
Large atelectasis	4 (0.2%)	0	24 (1.0%)	-
Large infiltrates	7 (0.3%)	2 (0.08%)	23 (0.9%)	12 (0.5%)
'Severe' pulmonary congestion	5 (0.2%)	1 (0.04%)	29 (1.2%)	-
'Severe' pleural effusion	3 (0.1%)	0	13 (0.5%)	5 (0.2%)
Pneumothorax or pneumomediastinum	2 (0.08%)	0	14 (0.6%)	5 (0.2%)
Malposition of oropharyngeal tube	1 (0.04%)	0	32 (1.3%)	19 (0.8%)
Malposition of intravenous lines	0	0	12 (0.5%)	9 (0.4%)
Malposition of intra-aortic balloon pump	0	0	1 (0.04%)	1 (0.04%)
Malposition of gastric tube	0	0	5 (0.2%)	3 (0.1%)
Malposition of drains	0	0	1 (0.04%)	1 (0.04%)
Total number of abnormalities	22	3	154	55
Total number of chest radiographs with abnormalities	19 (0.8%)	3 (0.1%)	142 (5.8%)	53 (2.2%)
Total number of patients with chest radiographs with abnormalities^b^	20 (2.7%)	3 (0.4%)	108 (14.3%)	48 (6.4%)

Predefined major abnormalities are summarized in Table 1. ^a^Absolute number of chest radiographs (% of all daily routine chest radiographs). ^b^Absolute number of patients (% of all patients with daily routine chest radiographs). -, not scored for; ICU, intensive care unit.

**Table 4:** Incidence of new expected and new unexpected predefined major abnormalities in 319 on-demand chest radiographs that were ordered as routine chest radiographs

Abnormalities	Expected abnormalities^a^		Unexpected abnormalities^a^	
	
	Abnormalities expected by the ICU physician	Abnormalities found by the radiologist	Unexpected abnormalities found by the radiologist	Abnormalities resulting in a change in therapy
Total number of abnormalities	137	8	29	11
Total number of chest radiographs with abnormalities	124 (38.9%)	8 (2.5%)	28 (8.8%)	11 (3.4%)
Total number of patients with chest radiographs with abnormalities^b^	89 (11.8%)	8 (1.1%)	27 (3.6%)	11 (1.5%)

Predefined major abnormalities are summarized in Table 1. ^a^Absolute number of chest radiographs (% of all daily routine chest radiographs). ^b^Absolute number of patients (% of all patients with daily routine chest radiographs).

**Table 5:** New and unexpected predefined major abnormalities on daily routine chest radiographs resulting in a change in management per admittance category

Abnormality	Diagnostic category (number of chest radiographs)				
	
	Medical (422)	General surgery (481)	Cardiopulmonary surgery (1251)	Neurosurgery (233)	Other (70)
Large atelectasis	-/8	-/3	-/12	-/0	-/1
Large infiltrates	1/3	2/5	9/13	0/2	0/0
'Severe' pulmonary congestion	-/1	-/2	-/25	-/1	-/0
'Severe' pleural effusion	1/4	3/5	1/3	0/1	0/0
Pneumothorax or pneumomediastinum	1/3	0/2	4/9	0/0	0/0
Malposition of oropharyngeal tube	5/8	3/6	8/15	3/3	0/0
Malposition of intravenous lines	1/2	1/2	7/8	0/0	0/0
Malposition of intra-aortic balloon pump	0/0	1/1	0/0	0/0	0/0
Malposition of gastric tube	1/2	0/0	2/3	0/0	0/0
Malposition of drains	1/1	0/0	0/0	0/0	0/0
Total (% of all daily routine chest radiographs in group)	11/32 (2.6%/7.6%)	10/26 (2.1%/5.4%)	31/88 (2.5%/7.0%)	3/7 (1.3%/3.0%)*	0/1 (0.0%/1.4%)*

Values are expressed as unexpected abnormalities resulting in a change in management (n)/all unexpected abnormalities per category (n); absolute numbers are given per diagnostic category. Predefined major abnormalities are summarized in Table 1. *P < 0.05 versus medical, general surgery and cardiopulmonary surgery. -, not scored for.
